# KH176 Safeguards Mitochondrial Diseased Cells from Redox Stress-Induced Cell Death by Interacting with the Thioredoxin System/Peroxiredoxin Enzyme Machinery

**DOI:** 10.1038/s41598-018-24900-3

**Published:** 2018-04-26

**Authors:** Julien Beyrath, Mina Pellegrini, Herma Renkema, Lisanne Houben, Svetlana Pecheritsyna, Peter van Zandvoort, Petra van den Broek, Akkiz Bekel, Pierre Eftekhari, Jan A. M. Smeitink

**Affiliations:** 1 Khondrion BV, Philips van Leydenlaan 15, 6525EX Nijmegen, The Netherlands; 2 Radboud Center for Mitochondrial Medicine, Radboud University Medical Center, Geert Grooteplein Zuid 10, 6500 HB Nijmegen, The Netherlands; 3 Department of Pharmacology and Toxicology, Radboudumc, Radboud Institute for Molecular Life Sciences, Grooteplein Zuid 28, 6525 GA Nijmegen, The Netherlands; 4 Inoviem Scientific SAS, Bioparc 3, 850 Boulevard Sébastien Brant, 67400 Illkirch-Graffenstaden, France

## Abstract

A deficient activity of one or more of the mitochondrial oxidative phosphorylation (OXPHOS) enzyme complexes leads to devastating diseases, with high unmet medical needs. Mitochondria, and more specifically the OXPHOS system, are the main cellular production sites of Reactive Oxygen Species (ROS). Increased ROS production, ultimately leading to irreversible oxidative damage of macromolecules or to more selective and reversible redox modulation of cell signalling, is a causative hallmark of mitochondrial diseases. Here we report on the development of a new clinical-stage drug KH176 acting as a ROS-Redox modulator. Patient-derived primary skin fibroblasts were used to assess the potency of a new library of chromanyl-based compounds to reduce ROS levels and protect cells against redox-stress. The lead compound KH176 was studied in cell-based and enzymatic assays and *in silico*. Additionally, the metabolism, pharmacokinetics and toxicokinetics of KH176 were assessed *in vivo* in different animal species. We demonstrate that KH176 can effectively reduce increased cellular ROS levels and protect OXPHOS deficient primary cells against redox perturbation by targeting the Thioredoxin/Peroxiredoxin system. Due to its dual activity as antioxidant and redox modulator, KH176 offers a novel approach to the treatment of mitochondrial (-related) diseases. KH176 efficacy and safety are currently being evaluated in a Phase 2 clinical trial.

## Introduction

The mitochondrial oxidative phosphorylation (OXPHOS) system plays a key role in cellular energy production by coupling the transfer of electrons to cellular respiration and ATP production^1^. The OXPHOS system is embedded in the inner mitochondrial membrane and is composed of five complexes (Complex I-V) and two electron carriers (ubiquinone and cytochrome *c*). It utilizes electrons from NADH (and FADH_2_) to reduce molecular oxygen (O_2_) to water with the concomitant production of ATP. Under physiological conditions, 0.25 to 11% of electrons escape the OXPHOS complexes depending on the animal species and respiration rates^2^. This occurs mainly at the level of Complexes I and III. These electrons react with environmental O_2_ to form the superoxide anion (O_2_^.−^), precursor of other reactive oxygen species (ROS). ROS comprise of both radical (*e*.*g*. superoxide and hydroxyl radical) and non-radical oxygen oxidants (*e*.*g*. hydrogen peroxide and hypochlorous acid), with different cellular production sites and biomolecule targets, including protein cysteine thiols^3^. Besides the well-known deleterious effects of ROS on macromolecules, ROS are central for mitochondrial and cellular signalling^4–6^. The regulation of intracellular ROS level depends on endogenous enzymatic systems, located in different cellular compartments, such as catalase, superoxide dismutases and peroxiredoxins, as well as non-enzymatic systems, such as glutathione (GSH), ascorbic acid and α-tocopherol^7,8^.

Defects of the OXPHOS system lead to a plethora of clinically variable signs and symptoms, together classified under the umbrella term of “mitochondrial diseases”^9^. The age of onset, disease course and disease progression are highly variable among mitochondrial diseases, ranging from fatal infantile multi-systemic lactic acidosis to single organ involvement^10^. The majority of mitochondrial patients lack therapeutic options aimed at stopping the disease progression or ultimately cure it, but only have access to supportive care^11,12^.

Despite the clinical heterogeneity of the mitochondrial diseases, from a cell biological perspective, OXPHOS system deficiencies show great similarities. Besides a lack of cellular energy, the alterations of the cellular redox state, increased ROS production and disturbances of the mitochondrial network and architecture are a general feature^13–15^. Genetic defects of subunits of the OXPHOS complexes can in fact result in increased ROS production leading to aberrations in the redox-controlled cell signalling, such as the thiol-based signalling, but also to irreversible damaging oxidation of macromolecules such as lipid peroxidation or protein carbonylation^16–18^. This cellular condition is often referred to as “oxidative stress” underlying the shift of the cellular redox balance towards a more oxidized environment^7,19^. Because elevated ROS levels and an imbalance in redox regulation are common pathological features in mitochondrial diseases they represent an attractive target for the development of treatments^20–22^.

Trolox, the water-soluble form of vitamin E, has been shown to have beneficial effects on cellular aberrations due to OXPHOS dysfunctions^23,24^. We have previously reported that an initial collection of newly generated Trolox-derivatives have improved antioxidant properties as compared to Trolox^25^, making these molecules attractive candidates for therapy development for mitochondrial diseases. We here present the optimization of this class of compounds towards the selection of the lead compound KH176, a clinical-stage oral drug candidate with a dual mode of action able to reduce cellular ROS and protect patient-derived cells from redox-induced cell death. We show that the activity of KH176 is depending on its redox potential and on proper functioning thioredoxin system/peroxiredoxin enzyme machinery.

## Results

### Increased ROS level and sensitivity to redox stress in Complex I deficient cells

**Figure 1:**
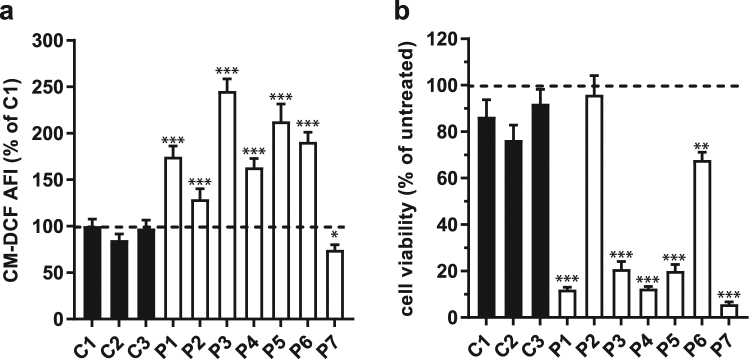
Oxidative stress in Complex I deficient primary human skin fibroblasts. (**a**) Basal ROS levels in 3 healthy control cell lines (C1 to C3) and 7 patient cell lines (P1 to P7) bearing mutations in different nuclear encoded Complex I subunits. Bar graphs represent the average of at least 3 independent measurements ± SD, and are normalized on C1 response. AFI = average fluorescence intensity. (**b**) Cell viability of the same panel of cell lines upon 24 h treatment with 100 µM BSO. Bar graphs represent the average of at least 3 independent measurements ± SD, and are normalized on the untreated condition per cell line. For statistical analysis each patient cell line was tested against the average of the 3 control cell lines. **p < 0.01, ***p < 0.001.

Oxidative stress is a common patho-mechanism described in primary mitochondrial diseases, but also in mitochondrial-related disorders such as Parkinson’s disease^14,26,27^. To develop a robust readout for this aspect of mitochondrial dysfunction that permits the screening of drug candidates, we have quantified both the basal cellular radical levels and cell sensitivity to redox perturbation in primary skin fibroblasts derived from patients with various pathogenic mutations in Complex I. Among a panel of seven patient-derived cell lines (P1 to P7), six of them displayed increased basal radical levels (Fig. 1a), as detected by the oxidation of the CM-H_2_DCFDA probe, as compared to three cell lines derived from healthy donors (all cell lines are described in Supplementary Table S1).

Five out of these seven patient-derived cell lines were sensitive to redox perturbation induced by 24 hours treatment with 100 µM Buthionine sulphoximine (BSO) (Fig. 1b and Supplementary Table S1). By inhibiting the gamma-glutamylcysteine synthetase, the rate limiting enzyme in the GSH biosynthesis, BSO treatment results in the depletion of GSH, an essential cofactor in numerous cellular redox reactions. The viability of the majority of the patient derived cell lines was severely affected, while cells from healthy donors remained viable after treated for 24 hours with 100 μM BSO (Fig. 1b and Supplementary Fig. S1). These results confirm oxidative stress as an important consequence of Complex I deficiencies. Although an increase in ROS level can lead to a redox imbalance, we noted that this correlation was not necessarily seen in all patient cell lines. For instance, while P7 cells have a basal ROS level even lower than the healthy controls, they are highly sensitive to GSH depletion. An opposite observation was made for P6 cells displaying a high ROS level but a low sensitivity to BSO (Fig. 1). This may reflect the impact of the different Complex I subunit mutations present in these cell lines, or may have other as yet unknown (genetic) causes. We therefore opted to use both tests in our drug screen.

### KH176 development and selection

**Figure 2:**
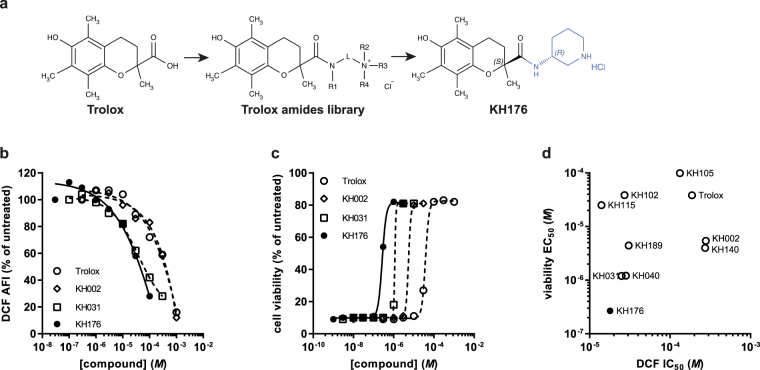
Screening strategy for the selection of KH176. (**a**) Chemical structures of the hit compound Trolox and the lead compound KH176. The scaffold used for the synthesis of the 226 new chemical entities is also shown. (**b**) P4 cell line was used to evaluate the ability of the newly synthesized Trolox-derivative compounds to reduce ROS levels after 72 h treatment with semi-logarithmic dilutions of the test compound, and (**c**) protect cell viability from BSO-induced toxicity. Four illustrative curves are shown for selected compounds with each data point representing the average of a triplicate measurement, normalized on the untreated condition. (**d**) IC_50_ (ROS assay) and EC_50_ values (Redox Stress Survival assay) were used to generate a correlation plot indicating KH176 with the highest overall potency among the screened compounds.

Based on initial findings we have previously reported that Trolox, the water-soluble form of vitamin E, and Trolox-derived antioxidants might be promising mitochondrial disease drug candidates^24,25^. We have now further optimized this initial set of compounds in order to improve their therapeutic potential and drug-ability. Starting from Trolox, we have synthetized a library of 226 new chemical entities (Patent WO2014011047 (A1)) by modifying the side chain on the carboxyl moiety while conserving the chromanyl group bearing the antioxidant capacity (Fig. 2a). The 226 newly developed compounds were tested on mitochondrial patient cells for their ability to scavenge cellular ROS (Cellular ROS assay, Fig. 2b) and protect patient-derived cells from redox perturbation (Redox Stress Survival assay, Fig. 2c). The applied screening strategy was an iterative process based on Structure Activity Relationship (SAR) studies, using P4 cells as a representative cell line displaying both pathological phenotypes. The calculated IC_50_ values from the ROS assay and the EC_50_ values from the Redox Stress Survival assay for all screened compounds were then plotted against each other in order to determine the compounds with the best overall performance. Figure 2d recapitulates the results of the most representative compounds, including the initial hit Trolox and the final lead KH176 ((S)-6-hydroxy-2,5,7,8-tetramethyl-N-((R)-piperidin-3-yl)chroman-2-carboxamide hydrochloride), and clearly depicts KH176 as the compound with the highest overall potency. These results furthermore show that the two pathways responded differently to the same drug. Indeed, while Trolox and KH002 demonstrated similar potency in the ROS assay (Trolox IC_50_ = 187 µM, KH002 IC_50_ = 278 µM), they markedly differed in the Redox Stress Survival assay (Trolox EC_50_ = 38 µM, KH002 EC_50_ = 5.4 µM). A similar pattern was observed for KH176 and KH031.

### KH176 pharmacology and metabolism

During pharmaco- and toxicokinetic studies (Tables 1 and 2, respectively) we identified, both in plasma and tissues of different animal species treated with KH176, a major metabolite with a quinone structure, referred to as KH176m. Due to its relative high proportion detected *in vivo*, with the ratio of KH176m/KH176 in systemic exposure going up to 0.97 in dogs (Table 2), we have identified and synthesized KH176m (Supplementary Fig. S2) and included this compound in further *in vitro* studies.

**Table 1:** Pharmacokinetic parameters of KH176 and KH176m in mice and rats, derived from a single dose pharmacokinetics and bioavailability study.

Animal species	Compound	Route	Dose (mg/kg)	T_max_ (hr)	^a^C_0(i.v.)_ C_max(p.o.)_ (ng/ml)	AUC_last_ (hr.ng/ml)	AUC_INF_ (hr.ng/ml)	T_1/2_ (hr)	CL (ml/min/kg)	V_ss_ (l/kg)	F^b^ (%)	Ranges (1–8 hours)			
												Liver to Plasma ratio	Heart to Plasma ratio	Skeletal muscle to Plasma ratio	Brain to Plasma ratio
Mouse	KH176	i.v.	2	—	543.3	180.6	190.8	1.4	174.7	9.5		10.5–29.3	4.3–28.3	8.7–20.1	1.0–25.0
		p.o.	10	0.25	414.1	617.7	623.5	n.d.	n.d.	—	68	5.7–12.1	2.4–5.7	0.8–5.2	0.4–0.8
	KH176m^c^	i.v.	—	—	359.3	140.0	146.9	0.5	226.9	8.2		29.0–57.1	6.1–11.6	8.2–20.5	b.d.
		p.o.	—	0.25	1061.8	1225.2	1260.8	n.d.	n.d.	—	—	12.8–94.1	2.1–14.6	4.4–33.8^d^	0.1–19.6
Rat	KH176	i.v.	2	—	344.1	509.9	517.8	2.6	64.6	7.0		n.d.	n.d.	n.d.	n.d.
		p.o.	10	0.67	594.2	1872.8	1933.6	n.d.	n.d.	—	74	n.d.	n.d.	n.d.	n.d.
	KH176m^c^	i.v.	—	—	59.7	150.2	206.1	2.3	163.7	29.1		n.d.	n.d.	n.d.	n.d.
		p.o.	—	0.5	396.4	1441.1	1503.2	n.d.	n.d.	—	—	n.d.	n.d.	n.d.	n.d.

Pharmacokinetic parameters of KH176 and its metabolite KH176m in plasma following single intravenous and oral dose administration of KH176 in male C57BL/6 mice and male Sprague Dawley rats. All numbers are the average of N = 3 animals. i.v. = intraveneous; p.o. = per os; C_max_ = the peak plasma concentration of a drug after administration; T_max_ = time to reach C_max_; AUC = area under curve; T_1/2_ = half-life; CL = clearance; V_ss_ = volume of distribution; F = oral bioavailability; ^a^back extrapolated concentrations for i.v. group; ^b^AUC_last_ considered for calculating the bioavailability; ^c^pharmacokinetic parameters for KH176m were calculated based on the KH176 dose; ^d^range at 1 hour, no concentrations were detected at 8 hours. n.d. = not determined; b.d. = below detection; — = not relevant.

**Table 2:** Toxicokinetic parameters of KH176 and KH176m in rats and dogs, derived from a 28-day repeat oral dose toxicology study.

Study	Species	Dose (mg/kg/dose BID)	Gender	Study Day	C_max_ (ng/ml)		AUC_0–24 h_ (ng.h/ml)			T_1/2_ (hours)	
					KH176	KH176m	KH176	KH176m	Ratio KH176m/KH176	KH176	KH176m
28-days repeat oral dose toxicity	**Rat**	25	M	1	979	92.7	10500	792	0.075	2.4	2.3
				28	1600	126	15600	993	0.064	5.6	13.3
			F	1	1360	85.9	12500	1140	0.091	2.6	4.5
				28	2270	123	19000	1410	0.074	3.2	6.2
		75	M	1	2860	187	33100	2640	0.080	4.5	3.5
				28	4210	265	46200	3100	0.067	n.d.	n.d.
			F	1	3050	203	42400	3090	0.073	8.4	30.9
				28	5340	305	76900	5120	0.067	6	12.9
		250	M	1	5890	312	94000	5170	0.055	n.d.	104.4
				28	11200	777	157000	10400	0.066	8.3	6.5
			F	1	6970	415	104000	7630	0.073	n.d.	n.d.
				28	13300	913	243000	17300	0.071	n.d.	n.d.
	**Dog**	10	M	1	2030	946	16400	13900	0.848	1.4	4.7
				28	2330	916	19000	12700	0.668	1.8	7.7
			F	1	1150	539	8840	7640	0.864	1.4	5.4
				28	1540	786	14600	10500	0.719	2	4.7
		25	M	1	4290	2610	42600	41200	0.967	1.8	11.1
				28	4540	2270	39900	38700	0.970	2	14.3
			F	1	4150	2200	41900	35400	0.845	1.8	n.d.
				28	5190	2170	40000	32900	0.823	2.5	n.d.
		62.5	M	1	10100	4020	91500	68000	0.743	1.8	12.9
				28	12600	5250	102000	85200	0.835	2.4	n.d.
			F	1	8150	3550	63100	57300	0.908	1.7	5.3
				28	10200	4990	85500	80200	0.938	2.3	12.2

Toxicokinetic parameters of KH176 and its metabolite KH176m in plasma following single and 28-days BID multiple oral doses administration of KH176 in Beagle dogs and Sprague Dawley rats. All numbers are the average of N = 3 animals (same animals for the two timepoints). M = male; F = female; p.o. = per os; BID = twice a day; C_max_ = the peak plasma concentration of a drug after administration; AUC = area under curve; T_1/2_ = half-life; n.d. = not determined.

Plasma pharmacokinetics and tissue distribution of KH176 and KH176m following a single intravenous (2 mg/kg) or per oral (10 mg/kg) dose administration were studied in male mice and rats. The results (Table 1) show that KH176 has a high oral bioavailability (F) of 68% and 74%, respectively. In addition, KH176 and its metabolite KH176m were detected in tissues such as brain, heart, muscle and liver. Both compounds had, due to the high metabolic rate in these species, a high plasma clearance with short half-lives in mice and rats.

Single- and multiple-dose toxicokinetics of KH176 and KH176m were studied in a 28-day repeat oral dose toxicity study (Table 2) in dogs and rats. In general, a dose-proportional increase in exposure of KH176 and KH176m was observed in both species from Day 1 to Day 28. Following repeated dosing, no marked accumulation in systemic exposure of KH176 and KH176m was observed at any dose level. KH176m to KH176 ratios in systemic exposure on Day 1 and Day 28 ranged from 0.67 to 0.97 in dogs and from 0.055 to 0.091 in rats. No gender difference in systemic exposure of KH176 and KH176m was observed.

### Characterization of KH176, KH176m and KH176i in cellular assays

**Figure 3:**
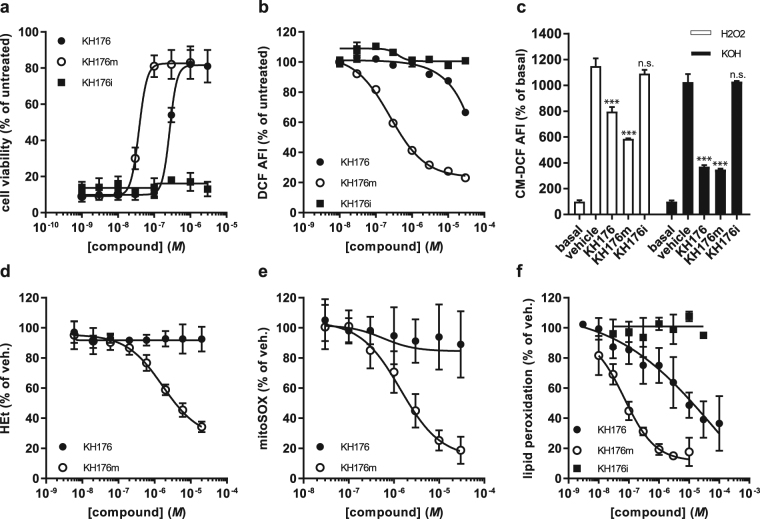
Effects of KH176, KH176m and KH176i in cellular assays. (**a**) Cell viability of P4 cell line treated for 24 h with 200 µM BSO in co-incubation with different concentrations of KH176, KH176m, or the redox-silent KH176i. EC_50_ KH176 = 2.7 × 10^−7^ M; EC_50_ KH176m = 3.87 × 10^−8^ M. (**b**) ROS assay performed on P4 cell line after 24 h compound treatment showing that KH176m is a more potent ROS scavenger than KH176 and KH176i is inactive. IC_50_ KH176m = 2.5 × 10^−7^ M. SD is indicated but is for most data points smaller than the symbol. **(c)** The P4 cell line was treated with 3 μM of KH176, KH176m or KH176i just before the addition of 100 µM H_2_O_2_ (white bars) or 1 mM KO_2_ (black bars). Neutralization of both H_2_O_2_ and O_2_^.−^ by KH176 and KH176m is shown by a decreased CM-H_2_DCFDA oxidation. Normalization was performed on the basal condition (no exogenous oxidant). Compounds effects were compared to vehicle; ***p < 0.001; n.s. = non-significant. **(d)** Cellular superoxide levels were detected using HEt. Basal levels were decreased in the presence of KH176m (EC_50_ KH176m = 1.7 × 10^−6^ M). KH176 had no effect. Results are the average of two independent experiments, SD is indicated. **(e)** Mitochondrial superoxide levels, detected with mitoSOX, could be decreased by KH176m (IC_50_ = 1.4 × 10^−6^ M). KH176 was much less potent. **(f)** CumOOH induced lipid peroxidation measured with bodipy 581/591 C11 could be inhibited by KH176 (IC_50_ = 6.4 × 10^−5^ M) and KH176m (IC_50_ = 7.1 × 10^−8^ M), KH176i had no effect. For all panels: unless otherwise indicated the data points represent the average of triplicate measurements and are normalized on the untreated condition, SD is indicated.

Figure 3a shows that both KH176 and KH176m could protect patient cell line P4 from BSO-induced death in the Redox Stress Survival assay. Interestingly, KH176m was more potent than KH176 with EC_50_ values of 38 nM and 269 nM, respectively. To strengthen these results additional cell lines were assessed. KH176(m) displayed similar cell protection to a BSO insult when using an extended panel of OXPHOS deficient cell lines (including different nuclear and mitochondrial DNA Complex I mutations, a complex V mutant and a complex III deficient cell line) (Supplementary Table S1), with EC50 values for KH176 ranging from 35 nM to 270 nM and for KH176m from 3.2 nM to 88 nM. This indicates that, like the sensitivity to BSO, the protection by KH176(m) is not exclusive for Complex I deficiency. Interestingly, KH176(m) was also active in patient cell line P7 displaying a lower ROS level than healthy controls (Supplementary Table S1 and Fig. 1). A redox-inactive form of KH176 (KH176i) was produced by substituting the hydroxyl function within the chromanyl group by a methoxy moiety that is resistant to oxidation (Supplementary Fig. S2), and as expected was unable to rescue cell viability.

When the three compounds were compared in the ROS assay (Fig. 3b), we observed that also in this assay KH176m was much more potent than KH176 (IC_50_ KH176 = 22.5 µM; IC_50_ KH176m = 255 nM), while KH176i was inactive. These results indicate that the activity of KH176 is indeed depending on its redox potential, since KH176i is inactive also in this assay. In order to investigate the type of ROS targeted by KH176 and KH176m, we used an artificial Radical Scavenging assay, where live cells are exposed to a burst of oxidants in the presence or absence of increasing concentrations of KH176, KH176m or KH176i. H_2_O_2_–induced intracellular radicals or O_2_^.−^ anions were triggered by adding to the cells 100 µM H_2_O_2_ or 1 mM KOH, respectively. The DCFH-DA (as well as CM-H_2_DCFDA) probe is known to be oxidized by these different free radicals^28,29^ and could therefore be used for their detection. The results depicted in Fig. 3c indicate that KH176 and KH176m, but not KH176i, were able to reduce the levels of both radicals, although the effect on superoxide induced by KOH was stronger.

The superoxide scavenging capacity of our compounds could be confirmed with the cytosolic superoxide specific HEt (dihydroethidium) dye. Oxidation of this dye under basal conditions without external stimuli could be inhibited with KH176m (Fig. 3d).

Although the formation of ROS inside the mitochondria will have deleterious effects also outside the organelles, since e.g. hydrogen peroxide freely diffuses over membranes, we also measured the ROS scavanging capacity of KH176(m) close to the source inside the mitochondria. For this we used the mitoSOX dye that due to its triphenylphosphonium moiety accumulates into mitochondria driven by the membrane potential. Without any external stimulus to the cells we could detect the mitochondrial ROS production after a 30 minutes incubation time. This ROS production was not significantly different in the different cell lines we used (both control and Complex 1 deficient, not shown). Preincubation with KH176m, and to a lesser extent KH176, resulted in a dose-dependent decrease of the mitochondrial mitoSOX signal (Fig. 3e).

Next, we investigated whether our compounds could also be active in scavenging ROS targeted at cellular membranes. For this we used the bodipy 581/591 C11 probe which due to its 11-carbon saturated fatty acid group localizes to the membrane fraction and changes its fluorescent spectrum upon oxidation. Cells were loaded with the probe in the presence of increasing amounts of KH176(m), and subsequently lipid peroxidation was induced with cumene hydroperoxide. Figure 3f shows that KH176(m) both were protecting the cells from lipid peroxidation, whereas KH176i again showed no activity. The IC_50_ of KH176m was about 1000-fold more potent than of KH176 in this assay. Similar results were obtained using a panel of fibroblast cell lines (not shown).

### KH176 protects from BSO-induced cell death without affecting the GSH level

**Figure 4:**
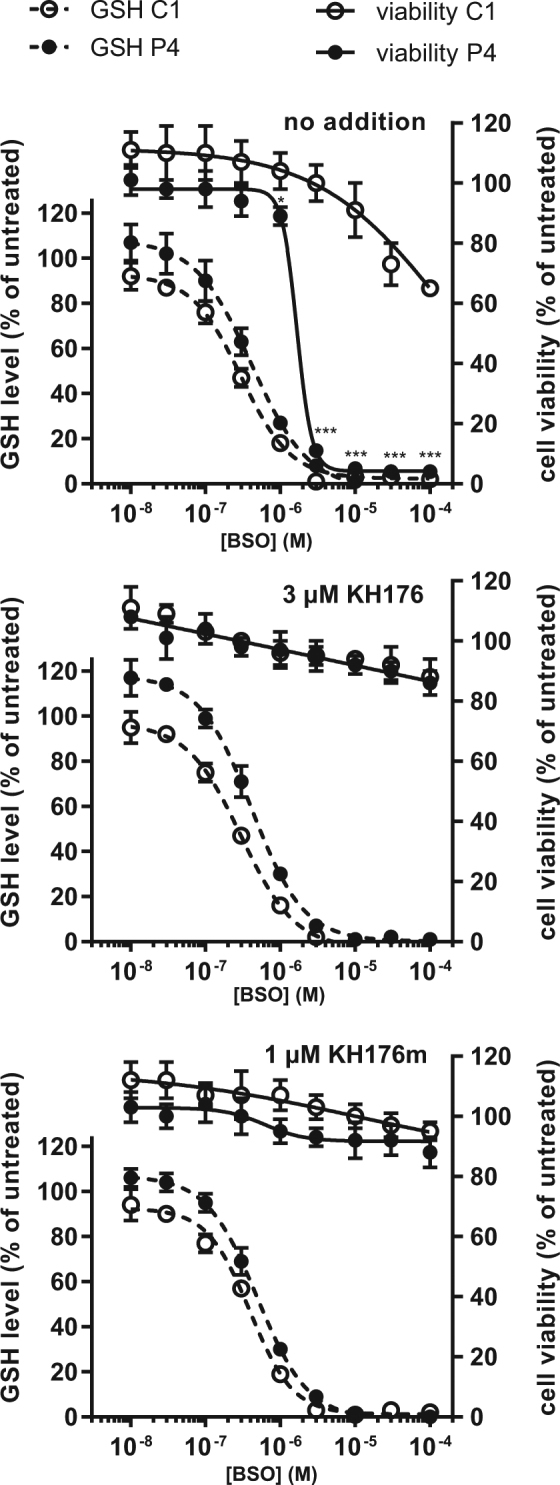
KH176 and KH176m can rescue BSO-induced cell toxicity without increasing the GSH level. GSH level and corresponding cell viability of C1 and P4 cell lines treated for 24 h with increasing concentrations of (**a**) BSO alone or (**b**) in co-incubation with 3 µM KH176 or (**c**) 1 µM KH176m. Dashed lines represent the average GSH level of at least 2 independent measurements ± SD, while solid lines show the parallel cell viability average value ± SD. Data are all normalized on the untreated condition. Statistical significance between C1 and P4 viability is indicated.

In order to understand the mechanism behind the protective effect of KH176 and KH176m in the Redox Stress Survival assay, the level of GSH was monitored in control and patient cells exposed to increasing concentrations of BSO and in the presence or absence of a fixed concentration of KH176 or KH176m. Figure 4 shows that while BSO-induced a similar dose-dependent decrease of GSH in both cell lines, this only affected the cell viability of the patient cells (Fig. 4a). Interestingly, while the viability of the patient cells was fully protected in the presence of 3 µM KH176 (Fig. 4b) or 1 µM KH176m (Fig. 4c), the level of GSH remained unaffected. This result indicated that the mode of action of KH176 and its metabolite KH176m did not involve competition with BSO-induced inhibition of gamma-glutamylcysteine synthetase nor alternative mechanisms to restore the GSH levels.

### Involvement of the TrxR-TRx-Prdx system in the KH176 mode of action

**Figure 5:**
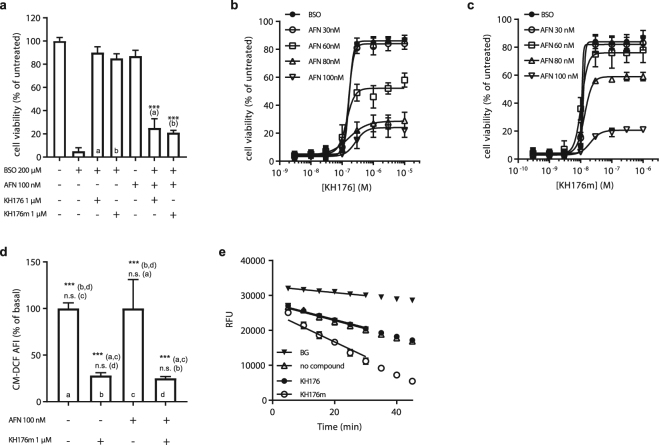
Involvement of the Thioredoxin System in the mode of action of KH176(m). (**a**) KH176 and KH176m can rescue P4 cells from BSO (200 µM)-induced death, their efficiency depends on an active Thioredoxin System since the presence of 100 nM AFN inhibits the rescue. ***p < 0.001 as compared to the indicated control. Increasing amounts of KH176 **(b)** or KH176m **(c)** rescue BSO (200 uM) treated cells, different concentrations of AFN affect the efficacy, but not potency of KH176(m). **(d)** the antioxidant activity of KH176(m) is not affected by inhibition of the Thioredoxin System by AFN (100 nM). ***p < 0.001 and n.s. = non-significant both as compared to the indicated bars. **(e)** KH176m but not KH176 enhances the Thioredoxin System/Peroxiredoxin-dependent consumption of NADPH. TrxR1, Trx1 and Prdx2 were incubated with NADPH and H_2_O_2_ in the presence or absence of 100 µM of KH176 or KH176m. The graph reports the fluorescence signal of NADPH over time. − = no KH compounds. For all panels: unless otherwise indicated the data points represent the average of triplicate measurements and are normalized on the untreated condition, SD is indicated. ***p < 0.001, n.s. = non-significant.

In mammalian cells, the cellular thiol redox homeostasis is controlled by two parallel systems: the GSH System and the Thioredoxin System^30^. Having ruled out an increase in GSH level upon KH176(m) treatment in our BSO-induced cell death model, we next evaluated the role of the Thioredoxin System in the redox-protecting mechanisms of KH176(m). The Thioredoxin Systems achieve their antioxidant function by transferring electrons to the peroxiredoxins that will use them to detoxify hydroperoxides in cells. We have used the well-characterized inhibitors of the thioredoxin reductases, auranofin (AFN) and aurothioglucose (ATG) that cause the inhibition of the entire TrxR-Trx-Prdx system^31^. Patient cells were incubated with a toxic concentration of BSO (200 µM) together with KH176 or KH176m that, as before, rescued the cells (Fig. 5a). Addition of 100 nM AFN resulted in a decrease of the capacity of KH176(m) to rescue the cells. We further observed in a dose response experiment (Fig. 5b and c) that the inhibition of thioredoxin system led to a reduced efficacy (% cell viability), but not potency (EC_50_) of KH176 and KH176m in the redox assay. This suggests that Auranofin and KH176(m) interact with different sites of the thioredoxin system. Similar results were obtained using ATG (not shown). These results established the involvement of the TrxR-Trx-Prdx system in the activity of KH176 and KH176m in the Redox assay.

We next assessed whether the same enzymatic system was required for the antioxidant property of KH176(m) in the ROS assay. Patient cells were therefore incubated for 24 hours with 100 nM of AFN together with KH176m, and ROS level was detected with the probe DCFH-DA (Fig. 5d). Addition of 1 μM KH176m reduced the DCFH-DA fluorescence as before (Fig. 3b). The addition of the thioredoxin reductases inhibitor AFN (or ATG, not shown) did not affect the capacity of KH176m to reduce the DCFH-DA signal. Similar results were obtained in other antioxidant assays using HEt, for cellular superoxide, and bodipy 581/591 C11 for lipid peroxidation (not shown). This result shows that, in contrast to the Redox assay, the antioxidant property of KH176(m) is not depending on the thioredoxin system.

To further delineate the mode of action of KH176(m), we examined their effects on the TrxR-Trx-Prdx enzyme machinery in a coupled enzymatic assay. The three recombinant enzymes were co-incubated with NADPH and H_2_O_2_ in the absence or presence of KH176 or KH176m. The reaction was followed in time by measuring the NADPH consumption. We found that KH176m, but not KH176, could significantly accelerate the consumption of NADPH, confirming an interaction of KH176m with the TrxR-Trx-Prdx system. (Fig. 5e). Further work is warranted to decipher whether KH176m is acting as a substrate or cofactor of the system.

### KH176 target deconvolution and in silico model

In order to identify the specific target of KH176m we have used the Nematic Protein Organization Technique (NPOT)^32^. NPOT is a label free proprietary technology (INOVIEM Scientific) used for the isolation and identification of specific macromolecular scaffolds involved in physiological or pathological conditions directly from human tissues. The technology is based on the Kirkwood-Buff molecular crowding and aggregation theory^33^ followed by mass spectrometry. We have studied the interactome of KH176m in different fibroblast cell lines and identified the peroxiredoxins as one of the top hits from this screen. Other identified proteins were part of the interactor network of peroxiredoxin, as shown on the string diagram (Supplementary Figure S3), and are still under study.

**Figure 6:**
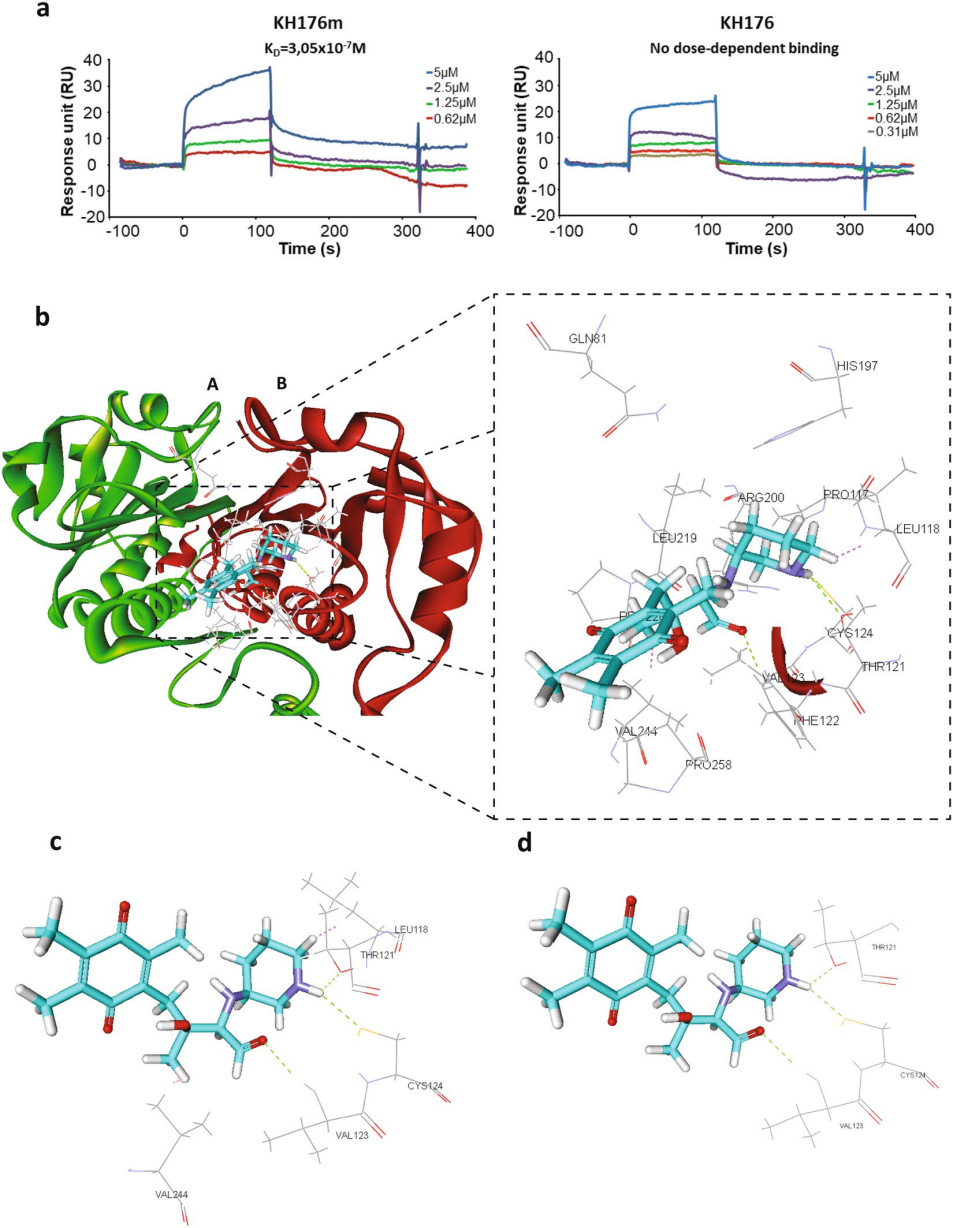
Interaction between KH176m and Peroxiredoxin. (**a**) Kinetic curves of KH176 and KH176m binding to immobilized human Prdx2 were obtained by Surface Plasmon Resonance. KH176m (left graph), but not KH176 (right graph), displayed a dose-dependent binding to Prdx2. (**b**) In this in silico model, KH176m interacts at a junction of the Peroxiredoxin 4 (Prdx4) dimer (A and B) in a pocket formed by 13 amino acids at a distance ≤10 Å. A nitrogen atom on the pyridine ring of KH176m interacts with Cys124 and Thr121 of the b monomer of Prdx4. On the same monomer, Val123 forms also a third hydrogen bond with the oxygen atom on the open ring of KH176m. KH176m forms also electrostatic interactions with Val244 and Leu118. (**c**) and (**d**) show different angles of the interaction between KH176m and Prdx4.

To assess KH176(m) binding to Prdx, we investigated the binding parameters and kinetics by Surface Plasmon Resonance. Prdx2 was immobilized onto the sensor chip via one of its lysines, and different concentrations of KH176m or KH176 were injected in the flux followed by a dissociation phase. KH176m, but not KH176, displayed a dose-dependent binding to peroxiredoxin (Fig. 6a). The affinity (Kd) calculated using Langmuir’s one site model was 0.305 µM for KH176m. Of note, no binding was observed between KH176m and another protein, CCDC101, identified in the NPOT experiment (Supplementary Figure S4).

This interaction of KH176(m) with the peroxiredoxins was further explored *in silico*. Since the 2-cystein Peroxiredoxins (Prdx1-4) have a high sequence homology, especially within the active sites, we used Prdx4 for the modelling since a high resolution crystal structure was available. Only KH176m was found to possibly interact at the junction of the Prdx4 dimers in a pocket formed by 13 aminoacids at a distance less than 10 Å. In the model the nitrogen on the pyridine ring of KH176m interacted with the peroxidatic Cys124 and with Thr121. Val123 also formed a third hydrogen bond with the oxygen on the quinone ring of KH176m. Also electrostatic interactions between KH176m and Val244 and Leu118 were possible (Fig. 6b–d). This *in silico* model was in accordance with our *in vitro* results showing that only KH176m had an effect on the enzymatic activity reaction.

To address the apparent discrepancy between the dependency in the Redox Stress Survival assay of both KH176 and KH176m on the TrxR-Trx-Prdx system on the one hand and the specificity for KH176m to stimulate this systems enzyme activity and to directly interact with the peroxiredoxins on the other hand, we measured the possible conversion of KH176 in cells. We found that after 24 hours incubation the conversion from KH176 into KH176m was quite substantial in cells with a ratio KH176m/KH176 of 0.48 (data not shown).

## Discussion

We aimed at improving the antioxidant properties of Trolox^25^ for the development of a potential treatment for mitochondrial disease patients, and have identified the small molecule KH176 as lead compound. Among more than 200 unique and novel chemical entities, KH176 was selected based on its physical-chemical properties, potency and efficacy. During pharmacokinetics and metabolism studies of KH176 *in vivo* in different animal species the formation of a major metabolite - KH176m - was reported, and therefore evaluated *in vitro* along with KH176.

We here show that the small molecule KH176, and its quinone metabolite KH176m, can counteract important cell biological consequences of Complex I dysfunctions being an altered cellular redox state and an increased ROS production^20^.

ROS and Redox are intertwined^34^ and it is therefore expected that higher ROS level will lead to redox imbalance. Interestingly, although on average our patients-derived cell lines display a higher ROS level and Redox sensitivity as compared to control cell lines, we observed at the individual level that the basal cellular ROS level and the sensitivity to BSO-induced GSH depletion are not directly correlated. Indeed, a patient cell line with a low ROS level had a high redox sensitivity (P7), or opposite (P6). In addition, we clearly show that the range of concentrations of KH176(m) required to protect the patient cells against BSO toxicity is at least a factor ten lower than the concentrations required to reduce pathological ROS level. This indicates that the protection of cells from a GSH-depletion by KH176(m) is not necessarily depending on its ROS scavenging property and that the compound has a dual mode of action, antioxidant and redox modulator. It is important to consider that the ROS level reported is quantified by the probe DCFDA and since the nature of the ROS detected with this probe is not fully clear we cannot exclude that using other ROS-reporting probes would directly correlate with Redox sensitivity. We have tried mitoSOX for mitochondrial superoxides detection but did not detect differences between control and patient cell lines (data not shown). Nevertheless, KH176(m) was found to be an effective antioxidant capable of scavenging ROS of different nature (H_2_O_2_, superoxide) and at different cellular locations (cytosol, membrane, mitochondrion).

Based on the current understanding of the effects at the cellular level of OXPHOS system defects, various new small molecules able to counteract such consequences are in preclinical development and some have reached the clinical development phase^12,35,36^. Among these molecules, only Raxone (INN idebenone) has so far obtained market approval^37^. This quinone compound with antioxidant properties, has been approved as treatment for LHON^38^, a mitochondrial disorder causing progressive visual loss. It increases the reduced ATP production due to Complex I deficiency in patients with LHON by transferring electrons directly to mitochondrial Complex III^39^. EPI743 is another quinone compound that, when tested in children with mitochondrial encephalomyopathies (Leigh Syndrome), could restore the level of reduced GSH in erythrocytes^40^ often found imbalanced in mitochondrial patients^41^.

Interestingly, in our Redox Stress Survival assay KH176 and KH176m could rescue the cells from BSO-induced cell death without restoring the BSO-induced decrease in GSH levels. This redox protecting ability of KH176(m) was found to extend to cell lines derived from patients with different OXPHOS system deficiencies.

When the thioredoxin system was pharmacologically inhibited, KH176(m) could not rescue the cells against BSO-induced toxicity, demonstrating that KH176(m) activity in the Redox assay was depending on the thioredoxin system, another major antioxidant and redox signalling cellular system. Of note, thioredoxin inhibition had no effect on the scavenging potential of KH176(m) in the ROS assay. This again highlights the independence of the two assays.

Thioredoxin system, composed of thioredoxin reductase, thioredoxin and peroxiredoxin, can efficiently reduce H_2_O_2_ into water using electrons from NADPH. Electrons are transferred between the 3 enzymes in a coupled oxidoreduction reaction. We have measured the effect of KH176(m) on the isolated thioredoxin system by following NADPH oxidation rate and observed that KH176m but not KH176 could increase the rate of NADPH consumption, indicative of an interaction with the system. We were unable to measure the rate of H_2_O_2_ reduction and therefore cannot conclude on the effect KH176m has on the peroxidase activity of the system.

By using an innovative target deconvolution approach, we have identified the peroxiredoxins as a predominant hit among the identified interacting partner of KH176m. Further interaction studies with Surface Plasmon Resonance, but also in silico modelling, confirmed the binding of KH176m, but not KH176, with peroxiredoxin. This highlights that KH176m, the quinone metabolite of KH176, is the active moiety in the redox assay, and that the effect of KH176 is dependent on its intracellular conversion to KH176. We have confirmed that KH176m was present in cells treated with KH176.

The peroxiredoxin enzymes are mainly described for their role in the detoxification of hydroperoxides in cells. One hypothesis is that KH176m increases the peroxidase activity of peroxiredoxins to reduce H_2_O_2_, acting as an electron shuttle. It could then be reduced directly by thioredoxin reductase bypassing thioredoxin. Indeed, quinones have previously been reported as substrates for thioredoxin reductase. However, there is recent evidence showing that Peroxiredoxins, but also Thioredoxins, can act as a relay in redox signalling, by acting as a sensor of H_2_O_2_ and passing the oxidized equivalent to other signalling proteins that are intrinsically less susceptible to direct oxidation by H_2_O_2_^42–47^. For instance, Prdx2, upon H_2_O_2_ sensing, can pass reducing equivalents to STAT3, a transcription factor involved in cell growth and apoptosis, attenuating thereby its transcriptional activity^48^. We envision that KH176m might in fact alter the transmission of such signal when interacting with the peroxiredoxins and therefore inhibiting the cell death signalling triggered by the BSO treatment. Further work is required to decipher the exact effect KH176m has on the peroxiredoxins, acting as a cofactor to increase its peroxidase activity or altering the peroxiredoxin-dependent redox signalling to other proteins.

Based on the detailed characterization of KH176, its mode of action, pharmacokinetics and toxicokinetics properties, a Phase 1 clinical trial in healthy adult male volunteers was performed (ClinicalTrials.gov Identifier NCT02544217). The study showed acceptable safety and good pharmacokinetic properties^49^. Ongoing clinical trial Phase 2 (ClinicalTrials.gov Identifier NCT02909400) and future Phase 3 studies will reveal whether and to what extend KH176 will be of clinically relevance for mitochondrial patients or even for other diseases and conditions with disturbed redox homeostasis.

## Material and Methods

### Primary Human Skin Fibroblasts Cultures

All primary human cell lines used throughout this study were received from RadboudUMC, Nijmegen, the Netherlands, and informed consent from donors or patient’s family members were obtained (see Supplementary Table S1). The cells were maintained in M199, HEPES (#22340020, Thermo Fischer Scientific) containing 10% FBS (#758093, Greiner Bio-one), 100 IU/ml penicillin and 100 µg/ml streptomycin (#30-002-CI, Corning) under a humidified atmosphere of 5% CO_2_ at 37 °C. The cells were passaged by trypsinization every 4–5 days until they reached the passage number 20, and then discarded. All compound and probe incubations were carried out under a humidified atmosphere of 5% CO_2_ at 37 °C.

### Cellular ROS Measurements in different cell lines

Cells were seeded at a density of 3 × 10^3^ cells/well into 96-wells black plates (#655090, Greiner Bio-one). After 24 h, the cells were loaded with 1 µM of the common ROS fluorescent indicator CM-H_2_DCFDA (#C6827, Thermo Fisher Scientific) in M199 without phenol red (#11043023, Thermo Fischer Scientific) for 20 min. The dye excess was washed with M199 without phenol red and CM-DCF fluorescence from live cells was measured on an automated bioimager (BD Pathway 855), by using a 20x objective and Ex. 488/10 nm, Em. 515LP nm. Total fluorescence per well was normalized on the number of cellular pixels/well.

### Compound screening on cellular ROS measurements

Cells were seeded at a density of 2 × 10^3^ cells/well into 96-wells transparent plates (#655101, Greiner Bio-one) and incubated for 24 h. The cells were then treated for 72 h (or shorter as indicated) with culture medium containing semi-logarithmic dilutions of the test compound. Alternatively, 3 × 10^3^ cells/well were seeded when cells were treated for 24 h only. At the end of the incubation period, cells were loaded with 40 µM DCFH-DA (#D6883, Sigma-Aldrich). in M199 without phenol red for 30 min. The excess of the dye was washed with ice-cold M199 without phenol red and the cells were lysed with 100 µl/well of ice-cold 0.2% Triton X-100 in M199 without phenol red after 10 min incubation in ice. Cellular debris was spun down by centrifugation at 2600 g, 5 min, +4 °C and 90 µl/well of cell lysate were transferred to a black 96-wells plate and DCF fluorescence was measured on a scanning microplate reader (BMG FLUOstar Omega; Ex. 480 nm, Em. 520 nm).

### Pharmacokinetics and toxicokinetics

Pharmacokinetics and toxicokinetics measurements were performed by the commercial Contract Research Organizations Sai Life Sciences and WuXi AppTec, respectively, according to their standard protocols and in accordance with relevant guidelines and regulations. C57 BL/6 mice, Sprague Dawley rats, and Beagle dogs were studied.

### Lipid peroxidation measurements

The cells were seeded at a density of 3 × 10^3^ cells/well into 96-wells black plates. After 24 h, the cells were coincubated with 500 nM bodipy 581/591 C11 (#D3861, Thermo Fisher) and different amounts of KH compound in M199 without phenol red. This medium was used in all subsequent washes and incubations. After 30 min. incubation the cells were washed twice and subsequently incubated with 30 μM cumene hydroperoxide (cumOOH). For background subtractions cells with the dye but without cumOOH were used. After 30 minutes incubation and two washes oxidized bodipy 581/591 C11 was quantified on an automated bioimager (BD Pathway 855), by using a 20x objective and Ex. 488/10 nm, Em. FURA/FITC. Total cellular fluorescence per well was normalized on the number of cellular pixels/well and normalized on the incubation with probe and cumOOH but without compound (vehicle set as 100%). Experiments were performed at least three times with 3 wells per experiment.

### Mitochondrial superoxide detection by mitoSOX oxidation

The cells were seeded at a density of 3 × 10^3^ cells/well into 96-wells black plates. After 24 h, the cells were incubated with different amounts of KH176(m) in M199 without phenol red. This medium was used in all subsequent washes and incubations. After 30 min. incubation the cells were washed and subsequently incubated 1 µM mitoSOX (#M36008, ThermoFisher). In our experiments a higher concentration of mitoSOX resulted in accumulation of the dye in the nucleus resulting in aberrant high fluorescence. After 10 min incubation the cells were washed twice and incubated for 30 min. to allow the accumulation of oxidized mitoSOX in the mitochondria. Total cellular fluorescence per well (BD Pathway 855, 20x objective, Ex. 488/10 nm, Em. 570LP) was normalized on the incubation with probe but without compound (vehicle set as 100%). Wells with cells but without mitoSOX were used for background correction. Experiments were performed at least three times with 3 wells per experiment.

### Cellular superoxide detection by Dihydroethidium (HEt) oxidation

The cells were seeded at a density of 3 × 10^3^ cells/well into 96-wells black plates. After 24 h, the cells were incubated with different amounts of KH176(m) for 24 h. For the cellular superoxide measurements cells were then washed with M199 without phenol red and incubated with HEt (#D11347, ThermoFisher) in this same medium for 10 minutes. After two washes with M199 without phenol red the cells were analyzed (BD Pathway 855, 20x objective, Ex. 488/10 nm, Em. 570LP). Total cellular fluorescence per well was normalized on the incubation with the probe but without compound (vehicle set as 100%). Experiments were performed at least three times with 3 wells per experiment.

### Redox Stress Survival Assay

Cells were seeded at a density of 4 × 10^3^ cells/well into 96-wells black plates and incubated for 24 h. To induce redox stress, semi-logarithmic dilutions of buthionine sulphoximine (BSO, #B2515, Sigma Aldrich) up to 100 µM were added to the cells in culture medium and incubated for 24 h. Cell viability was assessed by quantification of cellular conversion of Calcein AM into fluorescent Calcein. Briefly, after having discarded the BSO containing medium, cells were washed once with M199 without phenol red and loaded with 2 µM Calcein AM (#65-0853-39, Affimetrix eBioscience) in M199 without phenol red for 25 min. At the end of the incubation period, the excess of the dye was washed with M199 without phenol red and Calcein fluorescence was measured with a scanning microplate reader (BMG FLUOstar Omega; Ex. 480 nm, Em. 520 nm). For compound screening and target identification measurements cells were treated with 200 µM BSO alone or in co-incubation with semi-logarithmic dilutions of the test compound. Additionally, where indicated, increasing doses of Auranofin (AFN, #BML-EI206-0100, Enzo) or Aurothioglucose (ATG, #A0606, Sigma Aldrich) were co-incubated with BSO and the test compound. In all cases, cell viability was assessed 24 h after the treatment.

### Radical-Scavenging Capacity Assay

Cells were seeded at a density of 4 × 10^3^ cells/well into 96-wells black plates. After 24 h the cells were loaded with 5 µM CM-H_2_DCFDA in M199 without phenol red for 20 min. The dye excess was washed with M199 without phenol red and immediately after, cells were treated acutely with different concentrations of the test compounds in M199 without phenol red. To generate radicals, 100 µM H_2_O_2_ or 1 mM KO_2_ were injected to the compound-treated cells and CM-DCF fluorescence was measured every 2 min for 70 cycles on a scanning microplate reader (BMG FLUOstar Omega; Ex. 480 nm, Em. 520 nm). The area under the generated curves was calculated using the Origin 6.1 software (OriginLab).

### GSH measurements

Cells were seeded at a density of 4 × 10^3^ cells/well into 96-wells white plates (#655098, Greiner Bio-one). After 24 h the cells were treated with semi-logarithmic dilutions of BSO up to a concentration of 100 µM alone or in co-incubation with a fixed amount of the test compound. 24 h after the treatment, GSH levels were measured with the GSH-Glo™ Glutathione Assay (# V6911, Promega) according to the manufacturer instructions. Luminescence was measured on a scanning microplate reader (BMG FLUOstar Omega).

### TrxR1- Trx1- Prdx2 system biochemical assay

NADPH consumption measurements were performed according to the method described by Nelson *et al*.^50^ in the presence of TrxR1, Trx1 and Prdx2 (Novus Biological NBP1-44456, NBC1-18541 and NBC1-25855, respectively). The incubations contained TrxR1 0.5 µM, Trx1 5 µM, Prdx2 0.5 µM, H_2_O_2_ 50 µM, NADPH 75 µM in 25 mM potassium phosphate pH 7, 100 mM ammonium sulphate. The experiments were carried out in 96-wells black plates and in triplicate. Briefly, TrxR1, Trx1, and H_2_O_2_ were pre-incubated for 5 min at 25 °C for thermal equilibration. Upon addition of NADPH the initial NADPH auto-fluorescence was recorded for 2 min on a scanning microplate reader (PerkinElmer Enspire; Ex. 340 nm, Em. 482 nm) to insure that less than 10% of NADPH is oxidized in the absence of Prdx2. Ultimately Prdx2 was added to start the enzymatic reaction, which was monitored during 60 minutes. To test the effect of the compounds on NADPH consumption, KH176m and KH176 were added at 100 µM final concentration, after the addition of Prdx2. The NADPH fluorescence was followed in time. The assay was linear in time for at least 60 min. A parallel reaction without TrxR was performed to quantified non-specific NADPH oxidation and used for background correction.

### Intracellular conversion of KH176 into KH176m

Cells were seeded at a density of 125 × 10^3^ cells/well into 6-wells transparent plates (#657160, Greiner Bio-one) and incubated for 24 h. The culture medium was then replaced with fresh medium containing 3 µM KH176 and the cells were incubated for an additional 24 h. At the end of the treatment time, cells were washed twice with ice-cold PBS (#200120169, Thermo Fischer Scientific) before deprotenization with perchloric acid. The samples were then analyzed with standard LC-MS/MS system (Accela HPLC system, Thermo Fischer Scientific) equipped with a Zorbax Eclipse Plus C18 analytical column (Rapid Resolution HD 1.8 μm; 50 × 2.1 mm, Agilent, USA) and coupled with a UHPLC Guard Zorbax Eclipse Plus C18 pre-column (1.8 µm; 5 × 2.1 mm, Agilent). After separation the eluate was directly passed into a TSQ Vantage tandem mass spectrometer (Thermo Fischer Scientific) equipped with an electro-spray ionization source. Detection of the compounds was based on isolation of the protonated molecular ion, [M + H]+, and subsequent MS/MS fragmentations and selected reaction monitoring were performed. Proprietary compounds of Khondrion were used as internal standards.

### Identification of target proteins by Nematic Protein Organisation Technique

Nematic Protein Organisation Technique (NPOT) is a label free proprietary technology offered by INOVIEM Scientific and is used to isolate and identify specific macromolecular scaffolds implemented in basic or pathological situations directly from human tissues^32^. Specifically, human mitochondrial patient fibroblast homogenized at low temperature (4 °C) in the absence of any detergent, reducing agent or protease or phosphatase inhibitors. All dilutions and washes were performed in HBSS with osmolality, trace elements, vitamins and salts concentrations as close as possible to those of the interstitial medium or cellular cytoplasm (INOVIEM Scientific proprietary buffer). KH176 and KH176m (100 µM) were put in contact with the total tissue material. The macromolecular assemblies related to the specific ligands were then separated using a differential microdialysis system, based on liquid transitory pH gradient (pH 5–10) where the macromolecules (protein groups) can migrate in the liquid phase to their mean molecular zwitterion positions^51–53^. The gradually growing and migrating macromolecules will form nematic crystals to macromolecular heteroassemblies due to the molecular interactions between the ligand(s) and their targets. The heteroassemblies are then trapped in mineral oil (#1632129, Bio-Rad) and isolated and identified by mass spectroscopy directly in liquid.

### Surface Plasmon Resonance analysis

Prior to protein immobilization all proteins were dialyzed against HBS-E (10 mM Hepes, 150 mM NaCl, 3 mM EDTA) buffer that was previously filtered through a 0.45 µM membrane using dialyze slide according to the manufacturer instruction. Human recombinant proteins peroxiredoxin 2 (Novus Biological NBC1-25855) and CCDC101 (Abcam Ab124547) were immobilized on CM5 (carboxymethylated dextran covalently attached to a gold surface) using the amine coupling method according to manufacturer’s recommendation (Biacore, GE Healthcare). Briefly the CM5 surface was activated with the mixture of EDC/NHS. Recombinant proteins at dilution 1 µg/ml in a corresponding buffer i.e. Peroxiredoxin 2 and CCDC101 in sodium acetate buffer 100 mM (pH 4.9). The control channel was activated with EDC/NHS. All channels were thereafter saturated with 0.1 M ethanolamine solution. KH176 andKH176m were solubilized in HBSS at stock solution 1 mM. They were thereafter further diluted in HBSS to final concentrations 0.31e-6M, 0.62e-6M, 1.25e-6M, 2.5e-6M and 5.0e-6M and injected over the flow cells at a flow rate equal to 30 µl/h for 2 min, followed by a dissociation phase of 4 min. The sensor chip surface was regenerated with 1 mM HCl. The specific binding profiles were obtained after subtracting the response signal from the control empty channel and from blank-buffer injection. The specific binding was calculated using Biaeval 3 software (Biacore, GE Healthcare) with the postulate of 1:1 ligand/analyte interaction. Based on the Chi^2^ value in regard to the R_max_ (less than 10%) and the difference interval ±2.5 RU theoretic fitting compared to experimental results, best fitting algorithm between Langmuir or drifting base line was applied.

### In silico modeling of the KH176(m)-Peroxiredoxin 4 interaction

*In silico* modelling was performed using Accelrys discovery studio 2.1 software. Two PDB files for KH176 and KH176m, generated using the same program, and the PDB for Peroxiredoxin 4 (PDB 2PN8) were subjected to CHARMM force field. Thereafter, using the Libdock module within the Discovery Studio 3.5 software (DS3.5), KH176 or KH176m were tested for their interactions with the Prdx4 dimers. All generated files were subjected to standard dynamic cascade (Accelrys discovery studio module Simulation) and minimized with Steepest descents (Max = 500, RMS gradient = 0.1) and conjugate gradient (Max = 500, RMS gradient = 0.0001) as well as heating (steps 2000 kCal, time step = 0.001 sec, initial temperature = 50 kCal, target temperature = 300 kCal, adjusted velocity frequency = 50 Hz, saved results frequency = 100 Hz).

### Statistical analysis

Differences between healthy control and patient-derived cells, or vehicle and treated cells, were analysed using one-way ANOVA followed by Dunnett’s *post hoc* test. All analyses were performed using GraphPad Prism version 7.00 for Windows, GraphPad Software, San Diego, CA USA; www.graphpad.com.

### Data Availability

The datasets generated during and/or analysed during the current study are available from the corresponding author on reasonable request.

## Electronic supplementary material

supplemental file
