# Supplementary material for: KH176 Safeguards Mitochondrial Diseased Cells from Redox Stress-Induced Cell Death by Interacting with the Thioredoxin System/Peroxiredoxin Enzyme Machinery
Source: Sci Rep. 2018 Apr 26;8:6577. doi: 10.1038/s41598-018-24900-3 (PMC5920042; doi:10.1038/s41598-018-24900-3)
Supplement: Supplementary file 1 — supplemental file [file 41598_2018_24900_MOESM1_ESM.pdf]

KH176 SAFEGUARDS MITOCHONDRIAL DISEASED CELLS FROM REDOX STRESS-INDUCED CELL DEATH BY INTERACTING WITH THE THIOREDOXIN SYSTEM/PEROXIREDOXIN ENZYME MACHINERY

Julien Beyrath[1,#,*], Mina Pellegrini[1,#], Herma Renkema[1], Lisanne Houben[1], Svetlana Pecheritsyna[1], Peter van Zandvoort[1], Petra van den Broek[3], Akkiz Bekel[4], Pierre Eftekhari[4], Jan A.M. Smeitink[1,2]

Supplemental Information

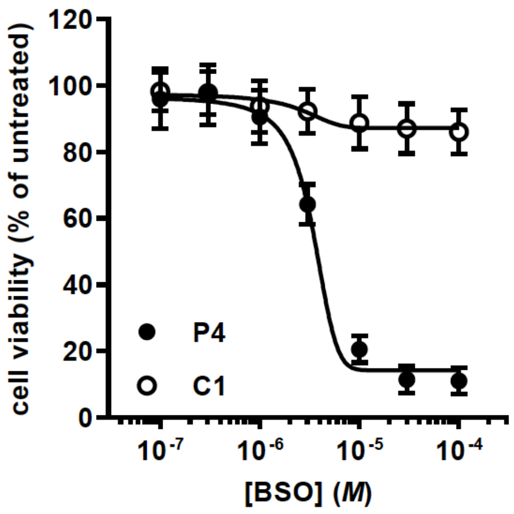

Supplementary Figure S1. Cell viability of C1 and P4 cell lines in response to 24 h treatment with semi-logarithmic concentrations of BSO up to 100 µM (P4 IC50=3.8 µM). Data points show the average value of triplicate measurements ± SD, normalized on the untreated condition per cell line.

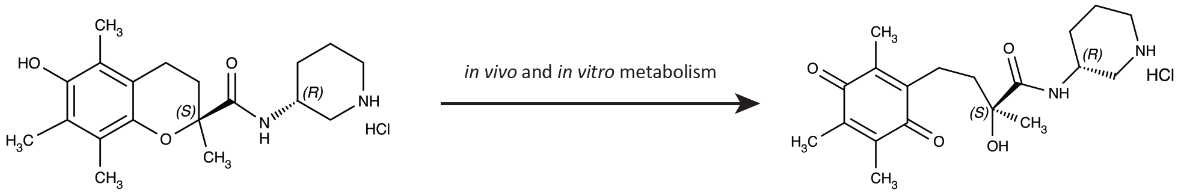

KH176 **KH176m**

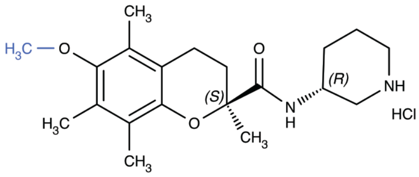

KH176i (Redox-inactive)

Supplementary Figure S2. KH176 structure and metabolism. The structure of KH176, its quinone metabolite KH176m and the redox-inactive form KH176i is reported.

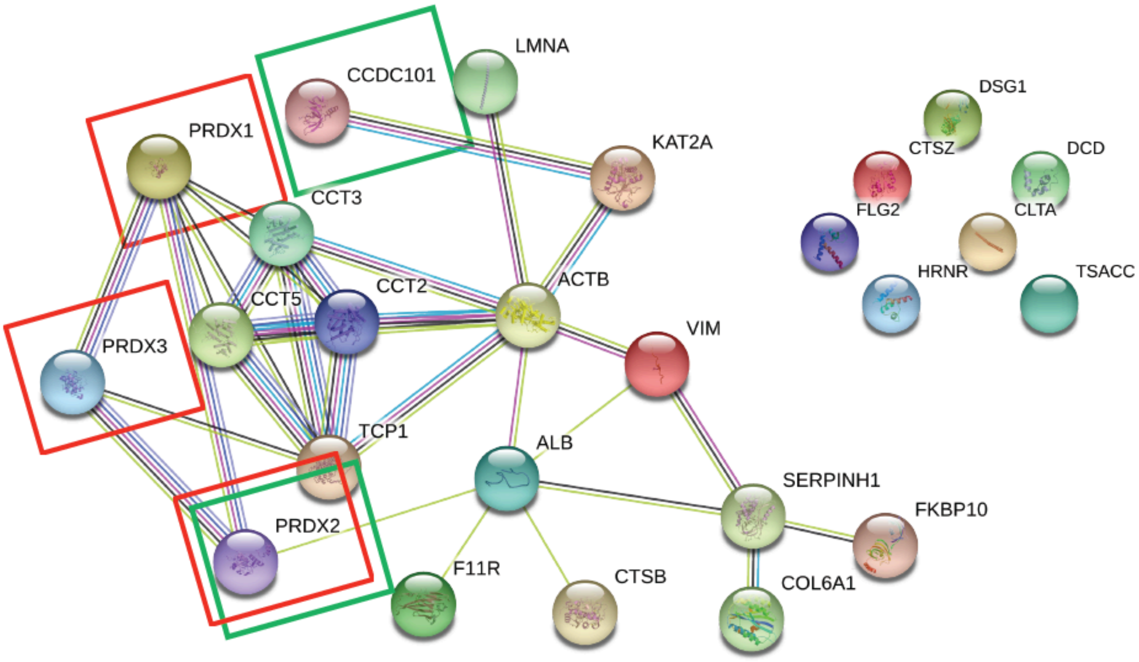

Supplementary Figure S3. String network diagram showing close interactors with Peroxiredoxins isolated with NPOT. Isolated proteins with no link to the network were considered as contaminants. Proteins framed in red square are from the same cluster. Proteins framed in green were selected for Surface Plasmon Resonance experiments.

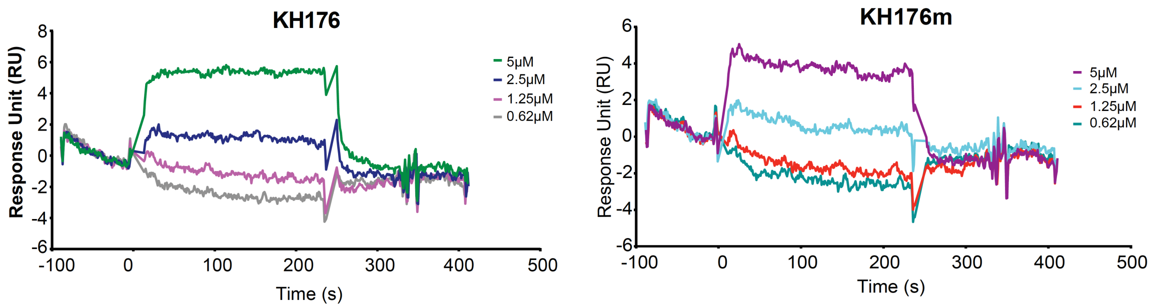

Supplementary figure S4. Kinetic curves of KH176 and KH176m binding to immobilized human CCDC101 were obtained by Surface Plasmon Resonance. KH176 (left graph) and KH176m (right graph) do not bind to CCDC101.

Code cell line	Genome	Affected OXPHOS Complex	Subunit	Gender	IC_{50} BSO (M)	EC_{50} KH176 (M)	EC_{50} KH176m (M)
**CONTROLS**							
**C1**	-	-	-	M	n.s.	-	-
**C2**	-	-	-	F	n.s.	-	-
**C3**	-	-	-	F	n.s.	-	-
**PATIENTS**							
**P1**	Nuclear	Complex I	NDUFS1	F	6.7E-6	7.2E-8	7.4E-9
**P2**	Nuclear	Complex I	NDUFS2	F	n.s.	-	-
**P3**	Nuclear	Complex I	NDUFS4	F	2.2E-5	6.6E-8	8.8E-8
**P4**	Nuclear	Complex I	NDUFS7	M	3.8E-6	2.7E-7	3.9E-8
**P5**	Nuclear	Complex I	NDUFS8	M	1.4E-5	5.3E-8	5.5E-9
**P6**	Nuclear	Complex I	NDUFS8	M	n.s.	-	-
**P7**	Nuclear	Complex I	NDUFV1	M	1.4E-6	1.8E-7	3.4E-8
**P8**	Mitochondrial	Complex I	ND1	M	2.5E-5	3.5E-8	3.2E-9
**P9**	Mitochondrial	Complex I	ND4	F	5.1E-6	1.4E-7	1.3E-8
**P10**	Mitochondrial	Complex I	ND6	F	8.6E-6	9.0E-8	1.3E-8
**P11**	Nuclear	Complex III	?	F	2.4E-5	3.8E-8	6.7E-9
**P12**	Nuclear	Complex V	ATP6	M	5.0E-6	1.4E-7	2.1E-8

Supplementary Table S1. Cell lines specifications. Detailed information on the human fibroblast cell lines used in the study, including their sensitivity to BSO dose response treatment (IC_{50} BSO) and the EC_{50}s of KH176 and KH176m in the Redox Stress assay. M=male; F=female. All donors were Caucasian. n.s.=not sensitive to BSO-induced toxicity; - = not relevant; ? = unknown.
